# Multi tyrosine kinase inhibitor dasatinib as novel cause of severe pre-capillary pulmonary hypertension?

**DOI:** 10.1186/1471-2466-11-30

**Published:** 2011-05-23

**Authors:** Jan K Hennigs, Gunhild Keller, Hans Jörg Baumann, Friedemann Honecker, Stefan Kluge, Carsten Bokemeyer, Tim H Brümmendorf, Hans Klose

**Affiliations:** 1 Centre for Pulmonary Hypertension, University Medical Centre Hamburg - Eppendorf, 20246 Hamburg, Germany; 2 Department of Internal Medicine II - Oncology, Haematology, BMT and Pneumology, University Medical Centre Hamburg - Eppendorf, 20246 Hamburg, Germany; 3 Department of Critical Care Medicine, University Medical Centre Hamburg - Eppendorf, 20246 Hamburg, Germany

**Keywords:** Pulmonary hypertension, drug induced, antiproliferative therapy, leukaemia, side effects

## Abstract

**Background:**

Pulmonary hypertension (PH) is a life-threatening disease with poor prognosis. Encouraging efforts have been made to target the main vasoproliferative aspects of the disease. Promising emerging therapeutics are tyrosine kinase inhibitors such as imatinib.

**Case presentation:**

Here, we discuss the relevance of previously published cases and add another well-characterised patient who developed pre-capillary PH under long-term therapy with the multi-tyrosine kinase inhibitor dasatinib approved for therapy of chronic myeloic leukaemia (CML) and Philadelphia chromosome positive acute lymphocytic leukaemia (mean time of all patients on dasatinib: 26 months). Hence, we discuss the possibility of dasatinib itself causing PH after long-term therapy and turn specialist's attention to this possible severe side effect.

At present, the true incidence of dasatinib-associated PH remains illusive and systematic data regarding haemodynamics are missing.

**Conclusion:**

We therefore recommend systematic screening of dasatinib-treated patients for pulmonary hypertension and subsequent collection of haemodynamic data.

## Background

Pulmonary hypertension (PH) is a severe and progressive, mainly vasoproliferative disease characterised by increased pulmonary artery pressure and vascular resistance eventually leading to right heart failure and death [1]. Different drugs have been identified to be causative of PH such as anorectic drugs which gained notoriety in the 1970s [2].

Dasatinib is a multi tyrosine kinase inhibitor approved for first and second line therapy of chronic myeloic leukaemia (CML) and Philadelphia chromosome positive acute lymphocytic leukaemia [3,4].

During the last months there have been two reports connecting dasatinib with the development of PAH [5,6]. Alarmingly, another patient was referred to our centre presenting with severe pre-capillary PH under dasatinib therapy.

Here, we report on this case and would like to turn attention to this possible severe side effect of dasatinib.

## Case presentation

A 70-year old male with chronic phase CML diagnosed in 1996 was changed to dasatinib therapy due to subsequent haematological progress under hydroxyurea combined with interferon alpha (1996-2002) and imatinib (2002-2004: 400 mg/day, 2004-2005: 800 mg/d). Dasatinib treatment with a dose of 70 mg bid was applied for 32 months. Side effects during this period were minor as the medication was generally tolerated well.

However, suddenly the patient developed tachy-dyspnea (25/min), transsudative, non-malignant pleural effusions (glucose 116 mg/dl; lactate dehydrogenase 188 IU/ml of effusions, serum lactate dehydrogenase 1073 IU/ml; protein content of effusions 31 g/l, serum protein content 67 g/l) and fatigue increasing within a few weeks.

Echocardiography showed highly increased right ventricular systolic pressure (RVSP) of 73 mm Hg. Invasive haemodynamic evaluation confirmed severe pre-capillary PH with consecutive right heart failure (details on prognostic factors and haemodynamics listed in Table 1). Clinically, the patient was assigned to WHO/NYHA functional class IV.

**Table 1:** Haemodynamic and prognostic data

	Time of presentation (/w dasatinib)	Time course under sildenafil (w/o dasatinib)			
Month:	*0*	*+1*	*+ 3*	*+ 5*	*+ 7*
**RVSP [mm Hg]**	73	51	-	17	-
**PAP**_**mean **_**[mm Hg]**	52	-	-	-	40
PVR [dyn*s/cm^**-5**^**]**	1250	-	-	-	356
**CO [l/min]**	1.7	-	-	-	4.7
**HR [/min]**	105	-	-	-	85
**proBNP [ng/l]**	27055	3037	1334	2076	-
**6MWD [m]**	0	308	458	-	-
**WHO/NYHA FC**	IV	II	I/II	II	II

RVSP: right ventricular systolic pressure; PAPmean: mean pulmonary artery pressure; PVR: pulmonary vascular resistance; CO: cardiac output; HR: heart rate; proBNP: brain natriuretic peptide propeptide; 6MWD: 6-minute walk distance; WHO/NYHA FC: World Health Organization/New York Heart Association functional class

As other underlying pathophysiological reasons were ruled out by serological tests, chest CT, scintigraphy of the lung and abdominal ultrasound, dasatinib was consequently discontinued. Normal wedge pressures at right heart catheterisation also excluded tyrosine kinase inhibitor-induced cardiomyopathy or other left heart diseases as possible underlying pathologies.

After discontinuation of dasatinib medication low-dose PAH-specific therapy with vasodilative phosphodiesterase-V inhibitor sildenafil (3 × 20 mg) was initiated. Acute symptoms relieved within days.

**Figure 1:**
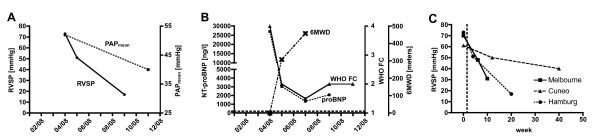
**Haemodynamics and prognosis factors of dasatinib-associated PAH**. Time courses of haemodynamics (Right ventricular systolic pressure, *RVSP *and mean pulmonary artery pressure, *PAPmean*, **A**) as well as exercise capacity (*6MWD*), WHO functional class and concentration of NT-proBNP (**B**) of the Hamburg patient are shown. Dashed horizontal line in (**B) **represents upper normal limit of NT-proBNP concentration (<197 ng/l). RVSP time courses of all three dasatinib-associated PH cases characterised so far are shown in (**C**). Vertical dashed line represents time of discontinuation of dasatinib treatment.

During the following 10 months prognostic parameters such as the N-terminal fragment of pro brain-natriuretic peptide (NT-proBNP), 6-minute walking distance (6MWD), RVSP, pulmonary artery mean pressure (PAP_mean_) and pulmonary vascular resistance (PVR) improved significantly (see Table 1). Additionally, the patient's subjective well-being advanced decisively which was also reflected by a functional class improvement to NYHA II (Figure 1).

### Does dasatinib itself trigger pre-capillary PH?

Pulmonary complications of dasatinib therapy have been reported ranging from pleural effusions to lung parenchymal affections [7]. In particular, pleural effusions caused by dasatinib, which are mostly exsudative due to clonal expansion of natural killer T cells, are well recognised and have been documented in various studies [3,7,8]. In addition to the EMEA data set [3], in a retrospective analysis of 138 patients receiving dasatinib in once or twice daily treatment schedules, pleural effusions of any grade were detected in 35% of the complete study population comprising chronic phase, accelerated phase and blast crisis [8].

Statistically significant, dose-dependent increase in RVSP was reported in a subgroup of 18 patients and dasatinib cessation led to normalisation of RVSP in 10 patients [8]. Dyspnoea, nausea, fatigue and oedema - unspecific yet classical symptoms found in PH - are among the most frequently observed unwanted dasatinib effects. Up to now, the extent of dasatinib-associated pre-capillary PH in contribution to these symptoms remains illusive since data regarding haemodynamics are lacking. However, all three detailed cases with dasatinib-related pre-capillary PH developed after a 2-year period of treatment (Figure 1C).

Our findings in combination with the two other published cases suggest a yet unknown association of pre-capillary PH with dasatinib [9]. Therefore, our patient was categorised into Dana Point class 1.3 that resembles drug-induced pulmonary arterial hypertension (PAH, [10]). Dana Point I represents the only class where PAH-specific drugs are labelled for. Hence PAH-specific therapy with low-dose sildenafil was started.

Dana Point class V, on the other side, combines all multifactorial or uncertain causes of PH and it explicitly includes chronic myeloproliferative diseases. Nevertheless, in this case connection of PH with myeloproliferative disease is also unlikely since, firstly, the CML was stable under dasatinib therapy for almost three years without acceleration until the end of dasatinib therapy. Secondly, a recent report showed a preferential association of myeloproliferative disease-associated PH with chronic thromboembolism [11], which was ruled out in our patient by CT scans and V/Q scintigraphy. In such case the use of sildenafil would have been off-label.

Taking into consideration that pleural effusions are found rarely in pre-capillary pulmonary hypertension another differential diagnosis has to be mentioned: pulmonary veno-occlusive disease (PVOD). PVOD, again, seems to be unlikely since it typically presents as non-responsive PH [12]. However, symptoms and RVSP of our patient rapidly improved after discontinuation of dasatinib and initiation of sildenafil therapy. Additionally, typical signs of PVOD such as ground glass opacity and septal thickening in CT scans or crackles and clubbing on examination [10] were absent in the presented case.

Most interestingly, although dasatinib also inhibits the platelet-derived growth factor receptor (PDGFR) pathway there may be a contrary effect on proliferative aspects of PAH compared to imatinib which has been shown to be an effective treatment improving PAH and animal survival as well as reversing pulmonary remodelling in rodent model systems, a series of case reports and a phase II trial [13-18]. Hence, a prospective randomised safety and efficacy trial is currently ongoing (IMPRES, *ClinicalTrials.gov*: NCT00902174).

As key mechanisms, imatinib reversed overexpression and increased phosphorylation of PDGFRβ in pulmonary arteries from rat models of pulmonary hypertension, inhibited PDGFR-related ERK1/2 activation in lungs of these animals thereby suppressing rat pulmonary artery smooth muscle cell (PA-SMC) proliferation and inducing PA-SMC apoptosis [13].

While dasatinib inhibits Bcr-Abl at significantly lower inhibitory concentrations (IC_50_) as compared to imatinib, its effect on c-kit and PDGFR are rather similar. In addition, however, and different from imatinib, dasatinib also inhibits the SRC family of kinases [19]. Whether this aspect of the compound is causally related to PH development remains unclear.

## Conclusion

There are emerging data that dasatinib could trigger drug-associated pre-capillary pulmonary hypertension. We therefore recommend the systematic screening and collection of haemodynamic data of dasatinib-treated patients.

## Consent

Written informed consent was obtained from the patient for publication of this case report. A copy of the written consent is available for review by the Editor-in-Chief of this journal.

## Competing interests

CB and THB have received travel grants, presentation fees and research grants form Bristol Myers Squibb. All other authors state no competing interests.

## Authors' contributions

FH, HJB, HK, THB and CB were involved in diagnostics and treatment of the patient. JKH, GK, HJB, SK and HK drafted the manuscript. All authors contributed to writing and editing the manuscript for important intellectual content. All authors read and approved the final manuscript.

## Pre-publication history

The pre-publication history for this paper can be accessed here:

http://www.biomedcentral.com/1471-2466/11/30/prepub
